# E-Cadherin and Gastric Cancer: Cause, Consequence, and Applications

**DOI:** 10.1155/2014/637308

**Published:** 2014-08-12

**Authors:** Xin Liu, Kent-Man Chu

**Affiliations:** Department of Surgery, The University of Hong Kong, Queen Mary Hospital, Pokfulam, Hong Kong

## Abstract

E-cadherin (epithelial-cadherin), encoded by the *CDH1* gene, is a transmembrane glycoprotein playing a crucial role in maintaining cell-cell adhesion. E-cadherin has been reported to be a tumor suppressor and to be down regulated in gastric cancer. Besides genetic mutations in *CDH1* gene to induce hereditary diffuse gastric cancer (HDGC), epigenetic factors such as DNA hypermethylation also contribute to the reduction of E-cadherin in gastric carcinogenesis. In addition, expression of E-cadherin could be mediated by infectious agents such as *H. pylori (Helicobacter pylori).* As E-cadherin is vitally involved in signaling pathways modulating cell proliferation, survival, invasion, and migration, dysregulation of E-cadherin leads to dysfunction of gastric epithelial cells and contributes to gastric cancer development. Moreover, changes in its expression could reflect pathological conditions of gastric mucosa, making its role in gastric cancer complicated. In this review, we summarize the functions of E-cadherin and the signaling pathways it regulates. We aim to provide comprehensive perspectives in the molecular mechanism of E-cadherin and its involvement in gastric cancer initiation and progression. We also focus on its applications for early diagnosis, prognosis, and therapy in gastric cancer in order to open new avenues in this field.

## 1. Introduction

Gastric cancer (GC) is the third leading cause of cancer-associated death worldwide [1]. More importantly, it is predicted that deaths from gastric cancer will rise from the 15th to the 10th cause of mortality from all causes globally by 2030 [2]. This underlies the emergency of breakthroughs in molecular mechanism of gastric cancer development to attenuate its harm. There are two main histological types of GC according to the World Health Organization (WHO) and the Laurén classifications, diffuse gastric cancer and intestinal gastric cancer, with distinct clinicopathological features [3]. Intestinal gastric cancer is more associated with environmental factors such as infection of* H. pylori*, high salty diet, smoking, and obesity [4, 5], while diffuse gastric cancer is composed by noncohesive cells and is more commonly observed in younger patients, with an obvious hereditary form. It has been reported that around 10% of the gastric cancer cases are familial clustering [6]. And the cases are defined to be hereditary diffuse gastric cancer (HDGC) by meeting the following criteria proposed by the International Gastric Cancer Linkage Consortium (IGCLC): (1) two or more documented cases of diffuse gastric cancer in first/second degree relatives, with at least one diagnosed before the age of 50 or (2) three or more cases of documented diffuse gastric cancer in first/second degree relatives, independently of age [7]. Interestingly, it has been reported that around 30% of the HDGC family harbor germline mutation of* CDH1* gene [8].

The* CDH1* gene locates in the human chromosome 16q22.1 and comprises 16 exons transcribed into a 4.5 Kb mRNA and encodes for E-cadherin [9]. E-cadherin is a calcium-dependent cell-cell adhesion molecule playing a crucial role in establishing epithelial architecture and maintaining cell polarity and differentiation [10, 11]. Germline mutations of* CDH1* gene predispose an individual to diffuse gastric cancer, and subsequent inactivation of the second allele of E-cadherin triggered by methylation, mutation, or loss of heterozygosity (LOH) leads to HDGC [12, 13]. Moreover, it has been revealed that cancer cells can disseminate to distant organs and dramatic alterations exist between cancer cells and extracellular-matrix components [14]. This leads to the attention that alterations in cell-cell adhesion and cell-matrix adhesion render tumor progression. Therefore, E-cadherin is pivotal in maintaining the epithelial architecture and cell polarity, while dysregulation of E-cadherin contributes to tumor invasion and progression [15], mainly diffuse gastric carcinoma in this review.

In addition to its role in cell-cell adhesion, E-cadherin and the cadherin-catenin complex could modulate various signaling pathways in epithelial cells, including Wnt signaling, Rho GTPases, and NF-*κ*B pathway. Therefore, dysregulation of E-cadherin promotes dysfunctions of these signaling pathways and influences cell polarity, cell survival, invasion, and migration in gastric carcinogenesis [16].

In this review, we summarize the function of E-cadherin and its associated signaling pathways, as well as the dysfunction of E-cadherin in gastric carcinogenesis, with an emphasis on diffuse gastric cancer. We also focus on the clinical applications of E-cadherin for diagnosis, prognosis, and therapy for gastric cancer.

## 2. Functions and Signaling Pathways of E-Cadherin

### 2.1. Structure and Functions of E-Cadherin

**Figure 1:**
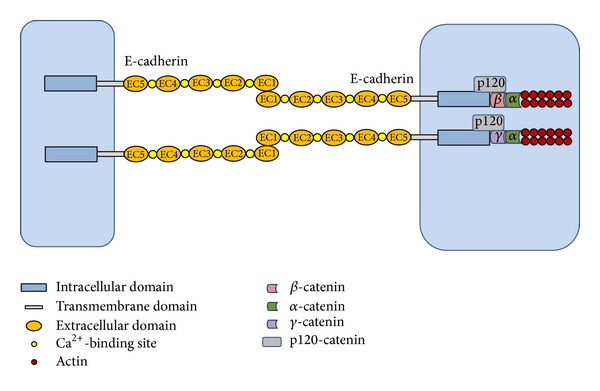
Schematic structure of E-cadherin and its binding to catenin proteins. The E-cadherin glycoprotein is composed of three major structural domains: an intracellular domain, a single transmembrane domain, and an extracellular domain comprising five tandemly repeated domains EC1–EC5. The intracellular domain of E-cadherin interacts with the catenins including *α*-, *β*-, *γ*-, and p120 catenin. The catenin anchors to the actin cytoskeleton, establishing cadherin-catenin complex. Conformation of E-cadherin is only stable upon Ca^2+^ binding to its extracellular motifs. Its stabilization at the cell membrane and accurate function occur by association to cytoplasmic p120-catenin.

The E-cadherin glycoprotein is composed of three major structural domains: a single transmembrane domain, connecting with a cytoplasmic domain, and an extracellular domain comprising five tandemly repeated domains called EC1–EC5, which are exclusive to cadherins [17]. The extracellular domain of E-cadherin is essential for cell-cell adhesion, as well as for the correct folding and homo/heterodimerisation of the proteins. The cytoplasmic domain of E-cadherin interacts with the catenins (*α*-, *β*-, *γ*- and p120 catenin) anchored to the actin cytoskeleton, establishing cadherin-catenin complex [18]. E-cadherin predominantly expressed at the membrane of epithelial cells, where it exerts cell-cell adhesion and suppresses invasion [10, 19]. Conformation of E-cadherin is only stable upon Ca^2+^ binding to its highly conserved, negatively charged extracellular motifs [20]. Its stabilization at the cell membrane and accurate function occur by association to cytoplasmic p120-catenin [21] (Figure 1). The stability of the cadherin-catenin complex, and its linkage to actin filaments, forms the core of the Adherens Junction (AJ), which is vital to inhibit individual epithelial cell motility and to provide homeostatic tissue architecture [22, 23]. Being a principal component of AJs, E-cadherin is essential for cell-cell contact of gastric epithelium. Hence, decrease of E-cadherin obviously contributes to dissemination of gastric cancer cell and further tumor progression.

### 2.2. Signaling Pathways Regulated by E-Cadherin

In addition to its role in cell-cell adhesion, E-cadherin is involved in a number of signaling pathways in carcinogenesis. As downregulation of E-cadherin in epithelial cells results in a reduced cell polarity and increased migratory and invasive-growth properties, loss of E-cadherin stimulates active signals that initiate epithelial mesenchymal transition (EMT) [24, 25]. Based on the various interaction partners of E-cadherin and the connection of the cytoplasmic cell-adhesion complex (CCC) to the actin cytoskeleton, a number of signaling pathways including Wnt signaling, Rho GTPases, and EGFR are thought to have an active part in the EMT process [22]. For the Wnt/*β*-catenin pathway, nonsequestered, free *β*-catenins may accumulate in the cytoplasm attributed to nonfunctional APC (adenomatous polyposis coli) or GSK-3*β* (glycogen synthase kinase 3*β*) [26]. High level of *β*-catenins in the cytoplasm subsequently translocates into the nucleus, binds to members of the TCF/LEF1 (Transcription Factor/Lymphoid enhancer-binding factor 1) family, and activates the expression of Wnt target genes, including* CD44, *c-*MYC*,* cyclin D1,* and* MMP7* [27]. Activation of these genes contributes to increased cell proliferation and tumor progression. Hence, it is supposed that E-cadherin expression can suppress Wnt/*β*-catenin signaling by sequestering *β*-catenin at sites of cell-cell contact. Evidence suggests that the mere presence of the E-cadherin cytoplasmic domain, rather than E-cadherin adhesive properties, is required to inhibit Wnt/*β*-catenin dependent gene expression [28]. In various cellular systems, it has been demonstrated that sequestration of *β*-catenin by E-cadherin can compete with the *β*-catenin/TCF-mediated transcriptional activity of the canonical Wnt signaling pathway [22].

Besides the Wnt signaling, another pathway frequently overexpressed in gastric cancer involves Rho GTPases, with Rho A, Rac1, and Cdc42 extensively studied [29, 30]. These molecules are known to play a critical role in cytoskeleton organization and cell motility [31]. It has been revealed that increased RhoA activity, which led to higher migration capacity, was induced by HDGC-associated E-cadherin missense mutations in the extracellular domain [32]. In addition, activation of RhoA through an E-cadherin dependent pathway involves the role of EGFR (epidermal growth factor receptor) [33]. Mutations at the E-cadherin extracellular domain may impair the interaction of E-cadherin and EGFR, lead to activation of EGFR, and further enhance cell motility through activation of RhoA [34]. Moreover, loss of E-cadherin and release of p120-catenin activate the Rac1-MAPK (mitogen-activated protein kinase) signaling pathway and promote transformed cell growth [35]. In summary, inactivation of E-cadherin leads to dysregulation of its associated signaling pathways and contributes to the EMT process and tumor progression.

Interestingly, the E-cadherin/catenin complex appears to possess the ability to downmodulate NF-*κ*B activity [36]. Importantly, NF-*κ*B regulates the phenotype of epithelial cells during inflammation, which has been shown instrumental to inflammation associated carcinogenesis, such as* H. pylori* infection in gastric cancer [37]. In mammals, the canonical NF-*κ*B activation pathway mainly applies to p65:p50 dimers, which are sequestered in a quiescent state in the cytoplasm by I*κ*B family members under steady state. On stimulation by a broad range of inflammatory mediators, including cytokines and microbial or endogenous danger-associated molecules, p65:p50 is released after I*κ*B phosphorylation by the IKK complex and subsequent degradation of this inhibitor. Finally, the heterodimer is translocated to the nucleus and activates the transcription of various target genes including* Bcl-2*,* IL-6*, and* TNF* [38]. The activation of these targets increases cell survival; reduces cell apoptosis; and contributes to inflammation associated cancer development. In a cellular system, it was shown that a forced overexpression of E-cadherin reduces NF-*κ*B activation, whereas loss of E-cadherin results in an increased activity of NF-*κ*B transcription factor [39]. In addition, NF-*κ*B suppression might result from a physical association with the E-cadherin/catenin complex [40]. Hence, the activation of NF-*κ*B through downregulation of E-cadherin provides convenience for the* H. pylori* infection associated gastric cancer development.

**Figure 2:**
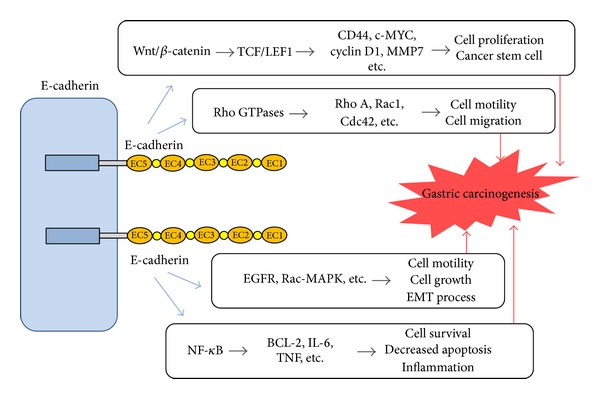
E-cadherin regulated signaling pathways involved in gastric cancer, including Wnt/*β*-catenin pathway, Rho GTPases, NF-*κ*B pathway, EGFR, and Rac-MAPK signaling. Activation of these pathways leads to increase in cell proliferation, decrease in cell apoptosis, cell migration, and inflammation associated gastric cancer development.

To summarize the above, dysregulation of E-cadherin leads to dysfunctions of E-cadherin-mediated signaling pathways, which alters cell polarity, increases cell survival, and promotes EMT process as well as cell invasion and migration [41]. These effects induce cancer initiation and progression, including gastric cancer (Figure 2).

## 3. Genetic Mutations and Variants of E-Cadherin in Diffuse Gastric Cancer

E-cadherin acts as a tumor suppressor and downregulation of E-cadherin is observed in various cancers [42]. Genetic mutation is one major mechanism for silencing tumor suppressor genes. Somatic mutations of* CDH1* have been identified in sporadic diffuse gastric cancer [43, 44], colorectal cancer [45], lobular breast cancer [46, 47], and ovarian cancer [48]. However, the report of familial gastric cancer without elevated rate of cancers in other organs suggested that alterations in the germline induced this inherited cancer [7, 49]. Germline mutation of* CDH1* was first reported in DNA extracted from lymphocytes of two patients with gastric cancer and four obligate carriers in New Zealand. The analysis of exon 2 to exon 16 of* CDH1* gene using the single stranded conformational polymorphism (SSCP) technique revealed a band shift in exon 7. Direct sequencing identified a* G* >* T* transversion in this exon. This mutation was not observed in 150 unrelated chromosomes [50]. Since then, genetic and germline mutations in* CDH1* gene have been analyzed widely in other populations with a family history of diffuse gastric cancer or patients with early onset diffuse gastric cancer (EODGC) (Table 1). These mutations lead to truncated proteins of E-cadherin, abnormal alterations of the E-cadherin's calcium binding sites, or increased its proteolytic degradation, which inactivate its functions. Inactivation of E-cadherin decreases cell-cell adhesion and induces aberrant alternations of E-cadherin-associated signaling pathways involving in cell proliferation, EMT process, and inflammation, and so forth. These aberrant changes trigger gastric cancer development.

## 4. Epigenetic Alterations and* H. pylori* Infection in Regulation of E-Cadherin Expression

Other than genetic mutations in* CDH1* gene to induce downregulation of E-cadherin, epigenetic factors also modulate the expression of E-cadherin. DNA methylation is a major type of epigenetic alterations and promoter hypermethylation exerts such modulation [55]. Germline mutations of* CDH1* gene predispose an individual to HDGC, and promoter hypermethylation frequently acts as the second hit to completely silence the gene. In sporadic diffuse gastric cancer, promoter hypermethylation of* CDH1* is more prevalent than mutation of the gene [56]. Moreover, it has been well studied that* H. pylori* infection is the strongest risk factor for gastric cancer development [57, 58]. Importantly,* H. pylori* infection modulates the promoter methylation status of abundant tumor suppressor genes in initiation and progression of gastric cancer, including* CDH1* (E-cadherin) [59].* CDH1* methylation seems to be an early event in* H. pylori* gastritis. It has been reported that* H. pylori* infection is associated with* CDH1* methylation in chronic gastritis patients [60]. Downregulation of E-cadherin by methylation was detected in precancerous lesions of gastric cancer, indicating that E-cadherin plays an important role in gastric cancer initiation [61]. More importantly,* H. pylori* is an independent risk factor associated with methylation of E-cadherin in nonlesion gastric mucosa from patients with dyspepsia [62]. The above evidence shows that DNA methylation is critical in modulating the expression of E-cadherin and such process can be regulated by* H. pylori* infection at the early stage of gastric cancer development. This provides potential clinical applications for diagnosis, prognosis, and therapeutic targets in gastric cancer, which will be discussed in the following part.

## 5. E-Cadherin in Clinical Applications for Gastric Cancer 

As E-cadherin plays a significant role in cell connection and its associated signaling pathways modulates epithelial cell fates and inflammation in gastric mucosa, inactivation of E-cadherin is critical in gastric cancer initiation and progression. Hence, evaluation of expression of E-cadherin or alterations in its encoded* CDH1* gene may provide promising applications for diagnosis, prognosis, or therapeutic targets for gastric cancer. The expression of E-cadherin is regulated by numerous factors including genetic mutations, DNA methylation, and* H. pylori* infection. It is assumed that detection of alterations in E-cadherin or its expression could reflect pathological conditions of stomach.

### 5.1. Soluble E-Cadherin as a Biomarker for Gastric Cancer

The extracellular domain of E-cadherin can be proteolytic cleaved by ADAMs (a disintegrin metalloproteinases), MMPs (matrix metalloproteinases), and KLK7 (kallikrein-related peptidase) under certain pathological stimulus such as* H. pylori *infection in the epithelial cells of stomach [63]. The proteolytic cleavage of E-cadherin generates an 80 kDa fragment which is released from the cell surface into circulation. This fragment of E-cadherin is termed as soluble E-cadherin [64]. Detection of soluble E-cadherin by ELISA (enzyme-linked immunosorbent assay) in circulation could indicate the status of gastric cancer. Two decades ago, it was first reported that soluble E-cadherin was elevated in serum of patients with gastric cancer compared with nontumor controls (*N* = 22) [65]. It was later confirmed in a larger sample size of gastric cancer patients (*N* = 81) [66]. This evidence indicates that soluble E-cadherin may serve as a prospective tumor marker that accurately reflects the progressive regeneration of E-cadherin at tumor sites. Furthermore, analysis of soluble E-cadherin in serum and cancer tissue provides hints for elucidating the mechanism of decrease of E-cadherin in cancer cells [67]. Moreover, high concentration of soluble E-cadherin in the serum of patients with gastric cancer predicts tumor T4 depth invasion and poor survival [68, 69], suggesting that E-cadherin could be applied as a valid prognostic marker for gastric cancer. It has also been revealed that high levels of soluble E-cadherin in serum 3 to 6 months after curative surgery could predict recurrence of gastric carcinoma [70]. The evidence mentioned above indicates that soluble E-cadherin could serve as a potential biomarker in diagnosis, prognosis, and tumor recurrence of gastric cancer.

### 5.2. Genetic Mutations of E-Cadherin (*CDH1*) for Clinical Management of Diffuse Gastric Cancer

As germ line mutation in E-cadherin (*CDH1*) gene was strongly involved in hereditary diffuse gastric cancer (HDGC), it was first proposed guidelines for clinical management of patients with familial diffuse gastric cancer in 1999. Later, the guidelines were updated in 2010 [7, 8]. These guidelines suggested that genetic counseling was essential for the evaluation and management of HDGC. Individuals with familial diffuse gastric cancer should take* CDH1* genetic screening and MLPA (multiplex ligation-dependent probe amplification) at a suggested age [71]. Individuals without* CDH1* mutation should take clinical surveillance by EGD (oesophagogastroduodenoscopy), while the ones with* CDH1* high risk missense mutations or truncating mutations was strongly recommended to take prophylactic gastrectomy and under close follow-up [8, 49]. The purpose of the guidelines is to establish a system to collect and collate data centrally, to combine the research process and clinical practice for a better patient management for the families affected by HDGC.

E-cadherin is encoded by the* CDH1* gene.* CDH1* gene is transcribed into a 4.5 Kb pre-mRNA. This pre-mRNA is processed to introns removal and exons connection, eventually generating distinct mRNA and protein of E-cadherin. The process of pre-mRNA to mature mRNA is called splicing. Splicing is regulated by* cis*-elements and* trans*-elements [72]. Abnormal alterations in these elements may lead to aberrant splice variants or abnormal expression, inducing dysregulation of maturation of E-cadherin. Abnormal maturation of E-cadherin leads to downregulation of E-cadherin and contributes to human hereditary diffuse gastric cancer (HDGC). Although still at the preliminary phase, it has been pointed out that targeting alternative pre-mRNA splicing such as the aberrant splice variants or their resulting products are potential therapeutic targets for HDGC [72]. It was reported that a germ-line splice site mutation (1135 ^∧^ IVS8 + 5del8ins5) of* CDH1* was identified in four members (father and three daughters) of a family with HDGC [73]. Besides gastric cancer [74, 75], splice site mutations of* CDH1* were also revealed in colorectal cancer and breast cancer [76, 77]. If the products generated from alternative pre-mRNA splicing of* CDH1* are identified, it will be possible to treat cancer patients more selectively. Through regulating the altered splice variants of the target gene rather than the whole target gene of an individual patient, personalized therapies are possible. However, one of the most important issues to be resolved is the development of a drug delivery system suitable for the therapeutics [78, 79].

### 5.3. *H. pylori* Infection and DNA Hypermethylation of E-Cadherin (*CDH1*) for Clinical Application for Gastric Cancer

*H. pylori* infection is involved in promoter hypermethylation of genes associated with the initiation and progression of gastric carcinogenesis [59]. Methylation of* CDH1* has been reported to be regulated by* H. pylori* infection in chronic gastritis and intestinal metaplasia patients, indicating that E-cadherin plays an important role in gastric cancer initiation [60, 62]. Importantly, eradication of* H. pylori* infection is able to reverse the hypermethylation status of* CDH1*, thus delaying or reversing* H. pylori* induced gastric carcinogenesis [60].

In addition to eradication of* H. pylori* infection, chronic aspirin use was indicated to be associated with a significantly lower methylation rates of* CDH1* gene (nonuser versus user 36.1% versus 10.8%, *P* = 0.005) in the gastric mucosa of* H. pylori* infection positive subjects [80]. Moreover, chronic NSAID (nonsteroidal anti-inflammatory drug) use was revealed to be inversely correlated with CIHM (CpG island hyper methylation) as an independent factor (OR = 0.18, 95% CI = 0.06–0.48) [81]. This suggests that NSAID can suppress CIHM of E-cadherin in the human gastric mucosa. Hence, chronic aspirin use or NSAID use may have suppressive role against methylation-related gastric carcinogenesis.

On the other hand, epigenetic alterations are reversible, drugs or chemical compounds with demethylating activity, such as 5-aza-2′-deoxycytidine (5-aza-dC), could be applied for patients with methylation of multiple tumor suppressor genes [82, 83]. Such therapy could also reverse the methylation status of* CDH1* and lead to reactivate the E-cadherin. Considering the adverse effects of 5-aza-dC, such as nonspecific demethylating and inducing genome wide hypomethylation, DNMT- (DNA methylation transferase-) targeted strategy has been proposed and may prove to be more effective than demethylating agents [84, 85].

## 6. Conclusions and Future Perspectives

Although alterations in E-cadherin and its expression may serve as promising biomarkers or therapeutic targets in gastric cancer, it should be aware of the potential pitfalls before applying for clinic. For example, it was reported that concentration of soluble E-cadherin did not elevate significantly in gastric cancer patients compared with healthy controls of a study in UK (*N* = 45) [86]. This might be attributed to the influence of age as it seemed that the concentration of soluble E-cadherin and other adhesion molecules increased as aging [87, 88]. Hence, comparison of serum levels of soluble E-cadherin should be considered in age-matched populations [89].

Moreover, a study of genetic analysis of* CDH1* gene in a Polish population (*N* = 86) indicated that mutations in* CDH1* did not contribute to familial diffuse gastric cancer in Poland [90]. In this study, the entire coding sequence of* CDH1* gene and exon/intron splice sites were applied for sequencing, but no pathogenic mutations were detected. This case suggested the need for mutation screening of other tumor suppressor genes, such as* TP53*, or screening for other genetic alterations, such as deletion, in familial diffuse gastric cancer lacking* CDH1* germline mutations [51].

On the other hand, attention should be paid for the promoting role of E-cadherin in tumor progression of several epithelial cancers proposed by recent studies. These include increased E-cadherin expression for supporting intravasation and tumor cell survival in inflammatory breast cancer [91, 92], E-cadherin associated mesenchymal to epithelial transition (MET) and activation of the Akt and MAPK signaling pathways in ovarian carcinoma [93, 94], and E-cadherin promoting cell proliferation and migration in a subset of highly aggressive glioblastoma [95, 96]. This process involved E-cadherin overexpression together with the E-cadherin associated specific signaling networks in the cytoplasm and nucleus [97]. This evidence indicates that the presence of E-cadherin as a tumor suppressor or oncoprotein depends on the specific cell context.

In summary, E-cadherin and its associated signaling pathways play important roles in maintaining functions of gastric mucosa. In contrast, dysregulation of these factors contributes to gastric cancer initiation and progression. Detection of the expression and aberrant alterations of E-cadherin are promising for clinical applications for diagnosis, prognosis and therapy for gastric cancer. However, it should be aware of the tumor promoting role of E-cadherin in specific cell context in order not to render development of other tumors by reactivation of E-cadherin for therapy of gastric cancer. Further studies of the functions of E-cadherin and the mechanisms of its tumor suppressing and tumor promoting roles are still in need for its wide application in clinic.

## Figures and Tables

**Table 1:** Genetic mutations of E-cadherin (*CDH1*) in diffuse gastric cancer in populations.

Population	Analytical methods	Mutations/variants	References
New Zealand	SSCP and sequencing	1008G>T in exon 7	[50]
Portuguese	PCR-SSCP and sequencing	1901C>T in exon 12	[51]
Chinese	PCR-DHPLC and sequencing	2253C>T in exon 14	[52]
Italian	SSCP, PCR and sequencing	163+37235G>A variant in intron 2	[53]
Korean	PCR, sequencing, and MLPA	1003C>T in exon 7	[54]

SSCP: single stranded conformational polymorphism; PCR: polymerase chain reaction; DHPLC: denaturing high-performance liquid chromatography; MLPA: multiplex ligation-dependent probe amplification.
